# The effects of an in utero exposure to 2,3,7,8-tetrachloro-dibenzo-*p*-dioxin on male reproductive function: identification of Ccl5 as a potential marker

**DOI:** 10.1111/j.1365-2605.2009.01020.x

**Published:** 2010-04

**Authors:** D Rebourcet, F Odet, A Vérot, E Combe, E Meugnier, S Pesenti, P Leduque, H Déchaud, S Magre, B Le Magueresse-Battistoni

**Affiliations:** * InsermU870, Oullins; † INRAUMR1235, Oullins; ‡ INSA-Lyon, RMNDVilleurbanne; § Université Lyon 1Lyon; ¶ Hospices Civils de LyonLyon; ** Inserm U863Lyon; †† BFA, CNRS EAC 7059, Université Paris 7France

**Keywords:** 2,3,7,8-tetrachlorodibenzo-*p*-dioxin, Ccl5/Rantes, in utero exposure, sperm count, testis, transcriptomic analysis

## Abstract

2,3,7,8-tetrachlorodibenzo-*p*-dioxin (TCDD) and dioxin-like compounds are widely encountered toxic substances suspected of interfering with the endocrine systems of humans and wildlife, and of contributing to the loss of fertility. In this study, we determined the changes in testicular gene expression caused by in utero exposure to TCDD along with the intra-testicular testosterone levels, epididymal sperm reserves, daily sperm production (DSP) and testis histology. To this purpose, female pregnant Sprague–Dawley rats orally received TCDD (10, 100 or 200 ng/kg body weight) or vehicle at embryonic day 15, and the offspring was killed throughout development. Hepatic Cyp1a1 gene expression was measured in the offspring to confirm the exposure to TCDD. The gross histology of the testes and intra-testicular testosterone levels were normal among the studied groups. Sperm reserves were altered in 67-day-old rats of the TCDD-200 group, but not in 145-day-old animals or in the other TCDD-exposed groups. Nonetheless, fertility was not altered in males of the TCDD-200 group, and the F2 males generated had normal sperm reserves and DSP. Microarray analysis permitted the identification of eight differentially expressed genes in the 4-week-old testes of the TCDD-200 compared with that of the control group (cut-off value ± 1.40), including the down-regulated chemokine Ccl5/Rantes. Inhibition of Ccl5/Rantes gene expression was observed throughout development in the TCDD-200 group, and at 67 and 145 days in the TCDD-100 group (animals of younger ages were not examined). Ccl5/Rantes gene expression was mostly confined in Leydig cells. F2 males generated from males of the TCDD-200 group had normal levels of Ccl5/Rantes in testis and Cyp1a1 in liver, which might indicate that Ccl5/Rantes is a marker of TCDD exposure in testis such as Cyp1a1 in liver. In conclusion, we demonstrated a decrease in Ccl5/Rantes RNA levels and a transitory decline in sperm reserves in the testes of rats of TCDD-dosed dams.

## Introduction

2,3,7,8-tetrachlorodibenzo-*p*-dioxin (TCDD) and dioxin-like compounds are widely encountered toxic substances suspected of interfering with the endocrine systems of humans and wildlife (Hotchkiss *et al.*, 2008; Diamanti-Kandarakis *et al.*, 2009). They form a large group of structurally related compounds of which the most toxic is TCDD (Van de Berg *et al.*, 2006). Dioxins are not intentionally produced, but are generated as undesired by-products in various industrial processes. They are persistent and, being fat soluble, tend to accumulate in higher animals, including humans. They are found in all environmental compartments although dioxin levels have been decreasing in the recent years in European countries, for example, to secure better protection of human health, in particular of children (Lundqvist *et al.*, 2006). Indeed, endocrine disrupters are suspected to be responsible for apparent changes seen over the recent decades, including congenital malformations, cancer and declining sperm counts. Genetic abnormalities are rare and cannot account for the rapid pace of the increase of reproductive disorders. The concept of testicular dysgenesis syndrome (TDS) was therefore proposed (Skakkebaek *et al.*, 2001). It enacts that the adverse changes, i.e. cryptorchidism, hypospadias, impaired spermatogenesis and testicular germ cell cancer, are inter-related and find common origins in foetal life or childhood. Supporting the concept of environmental influence was the demonstration in rats that foetal exposure to high doses of dibutyl phthalate causes a TDS-like phenotype (Fisher *et al.*, 2003; Sharpe & Skakkebaek, 2008).

Today, dioxins are found in all humans, and exposure to dioxins in the general population of the European Union, for instance, is at a level where subtle health effects might occur and it is, therefore, of utmost importance to improve the assessment of health risk (Gies *et al.*, 2007). Dioxins are not genotoxic, but cause a broad spectrum of adverse effects including hepatotoxicity, immune system suppression, developmental toxicity and skin defects. Meanwhile, there are still controversial data regarding the impact of dioxins on the male reproductive function, specifically in case of maternal exposure. Parameters previously investigated included at least measurement of testosterone levels, testis weight and sperm reserves in the offspring of dosed rat or mouse dams. Declining epididymal sperm reserves were often (Mably *et al.*, 1992; Gray *et al.*, 1995; Theobald & Peterson, 1997; Faqi *et al.*, 1998; Simanainen *et al.*, 2004) but not systematically (Wilker *et al.*, 1996; Ohsako *et al.*, 2001, 2002; Ikeda *et al.*, 2005; Bell *et al.*, 2007a) reported. Besides, molecular mechanisms have not been clarified.

2,3,7,8-tetrachlorodibenzo-*p*-dioxin mediates its toxicity by binding to the aryl hydrocarbon receptor (AhR) and subsequent alteration of the expression of target genes which exhibit dioxin response elements in their promoter moiety, including the cytochrome Cyp1a1 (Mimura & Fujii-Kuriyama, 2003; Barouki *et al.*, 2007). Therefore, microarray analyses have been conducted to select the most up- and down-regulated genes in target tissues. The liver has been mostly evaluated because of the strong hepatotoxicity of TCDD, and major genes related to cholesterol biosynthesis, glucose metabolism and lipogenesis consistent with complementary histopathology have been identified (Fletcher *et al.*, 2005; Sato *et al.*, 2008). Few data are available regarding the testicular transcriptome in rats exposed to TCDD. Two genes of germ cell origin have been identified using a representational difference analysis, and their expression was found to be down-regulated in the testes of adult mice or rats exposed to high doses of TCDD (Kuroda *et al.*, 2005; Yamano *et al.*, 2005). In studies investigating the consequences of a maternal exposure to dioxins, foetal pituitary gonadotrophin was defined as an initial target of dioxin indirectly impacting testicular steroidogenesis (Mutoh *et al.*, 2006), although direct testicular effects have also been described (Adamsson *et al.*, 2008).

In this study, we determined the changes in testicular gene expression caused by an in utero exposure of female pregnant Sprague–Dawley rats to TCDD along with the measurement of the intra-testicular testosterone levels, epididymal sperm reserves, daily sperm production (DSP) and testis histology in the offspring throughout development.

## Materials and methods

### Experimental design

Time pregnant Sprague–Dawley females of embryonic day 12 were purchased from Janvier’s Breeding (Le Genest, France). They were housed individually in plastic cages with food (Altromin 1310; Genestil, Royaucourt, France) and water provided ad libitum at 23 °C and a 12 : 12 photoperiod. Relative humidity was 50 ± 10%. Animals were randomly assigned to treatment groups. Dams were allowed 3-day acclimatization and were given one oral dose of 2,3,7,8-TCDD (ref. ED-901-C) (LGC Promochem, Molsheim, France) in sesame oil on embryonic day 15. Control animals received sesame oil. Maximal volume of gavages was 0.7 mL/animal. Aliquots containing various doses of dioxins were assayed by Dioxlab (Dioxlab, Saint-Maurice, France) to ascertain doses given to the animals. A total of 24 animals were used in the reported experiments. Of these animals, nine dams received sesame oil (control group), nine dams received one dose of TCDD 200 ng/kg body weight (bw) (TCDD-200 group), three dams received one dose of TCDD 100 ng/kg bw (TCDD-100 group) and three dams received one dose of TCDD 10 ng/kg bw (TCDD-10 group). The outcome of gestation, number of pups, sex ratio and weight was recorded. Pups were not individually identified in the litters, for bw. Male fertility of the TCDD-200 group (TCDD-F1 males) was assessed using two virgin females per male. It was compared with the fertility of the control-F1 males. Males originating from TCDD-F1 and control-F1 males were considered as TCDD-F2 and control-F2 males respectively. TCDD-F2 males have not been exposed in utero to TCDD. Rats were killed by cervical dislocation under CO_2_ anaesthesia at various ages. Testes and epididymes were dissected, weighed, prepared for morphological studies or frozen in liquid nitrogen and stored at −70 °C until processing for RNA analysis. Intra-testicular content of testosterone and 4-androstenedione was measured by radioimmunoassay as described elsewhere (Rinaldi *et al.*, 2001). Epididymal sperm reserves and DSP were measured as described elsewhere (Robb *et al.*, 1978). Livers were dissected for RNA purpose. All experiments were conducted with the approval of the local committee on animal care, and in accordance with the European guidelines (86/609/CEE).

### Histological analysis and immunofluorescence

For histological and immunohistochemical analyses, tissues were fixed at 4 °C in 0.1 m phosphate buffer, pH 7.4, containing 4% formaldehyde plus 10% picric acid for at least 24 h, dehydrated in a graded series of ethanol and paraffin-embedded using standard protocols. Sections 5 μm in thickness were stained with the periodic acid-Schiff (PAS)–haematoxylin technique. For immunohistochemical analyses, paraffin-embedded tissues were deparaffinized in xylene, rehydrated in graded ethanol solutions and endogenous peroxidase activity was blocked with 0.3% hydrogen peroxide in methanol for 20 min. The sections were sequentially incubated overnight at 4 °C with the anti-Ccl5/Rantes primary antibody (sc-1410; diluted 1/100) (Santa-Cruz Biotechnologies Inc., Santa Cruz, CA, USA), and anti-goat Ig-Alexa Fluor 555 secondary antibody (diluted 1/1000) (Invitrogen France, Cergy-Pontoise) for 1 h at room temperature. Leydig cell identity was revealed using the anti-3β-hydroxysteroid dehydrogenase (3beta-HSD) antibody (diluted 1/1000; overnight incubation at 4 °C) (provided by Dr I Mason, Reproductive and Developmental Sciences Division, Edinburgh, UK) followed by incubations with anti-mouse Ig-Alexa Fluor 555 secondary antibody (diluted 1/1000; 1 h at room temperature) (Invitrogen). Fluorochrome-labelled sections were mounted in vectashield containing DAPI for nuclei visualization (Vector Laboratories Canada, Burlington, ON, Canada). Slides were analysed with Zeiss Axiovert epifluorescence microscopes (Carl Zeiss, New York, NY, USA), all connected to a digital camera (Spot RT Slider, Diagnostic Instruments, Sterling Heights, MI, USA).

### Microarray analysis

Total RNA was extracted from testes and livers recovered from the control and TCDD groups of rats using Rneasy mini kit (Qiagen, Courtaboeuf, France). RNA integrity was determined with the Agilent 2100 Bioanalyzer and RNA 6000 Nano Kit (Agilent Technologies, Massy, France). For microarray analysis, a pool of control testes RNA originating from three different males of three different dams was used as a common reference and compared with three testes originating from three different TCDD-200 F1 males of three different dams. One microgram of total RNA was amplified with the Amino Allyl MessageAmp II aRNA kit (Ambion, Austin, TX, USA) according to the manufacturer’s instructions. This mRNA amplification procedure is well validated and it has been demonstrated that it does not distort the relative abundance of individual mRNAs within an RNA population (Wang *et al.*, 2000). Fluorescent probes were synthesized by chemical coupling of 5 μg of aminoallyl aRNA with cyanine (Cy)3 or Cy5 dyes (GE Healthcare Biosciences, Orsay, France). After purification with RNeasy Mini Kit (Qiagen), probes were fragmented with 25X RNA Fragmentation Reagents (Agilent Technologies) and hybridized with 2X Agilent Hybridization Buffer (Agilent Technologies) to *Rattus Norvegicus* opArray (Operon Biotechnologies GmbH, Cologne, Germany) in an Agilent oven at 67 °C for 16 h, following a dye swap experimental procedure to correct for gene-specific dye bias (Churchill, 2002). Microarrays were washed and scanned with a Genepix 4000B scanner (Molecular Devices, Sunnyvale, CA, USA). TIFF images were analysed using genepix pro 6.0 software (Molecular Devices). Signal intensities were log-transformed and normalization was performed by the intensity-dependent Lowess method. To compare results from different experiments, data from each slide were normalized in log-space to have a mean of zero using cluster 3.0 software (http://rana.LbL.gov/EisenSoftware.htm). Only spots with signal to noise ratio above two were selected for further analysis. Data were analysed using the significance analysis of microarray procedure, with a false discovery rate of 5% (Tusher *et al.*, 2001). Microarray data are available in the GEO database under the number GSE13838.

### Quantitative polymerase chain reaction (Q-PCR)

Quantitative polymerase chain reaction was used for validation of the microarray procedure and study of gene expression levels of testicular Ccl5/Rantes and hepatic Cyp1a1 in rats from the different experimental groups, including the offspring of the dosed dams and the males of the F2 generation. Each RNA sample used for Q-PCR was prepared from rats that originated from different dams. Briefly, first-strand cDNAs were synthesized from 1 μg of total RNA in the presence of 100 U of Superscript II (Invitrogen, Eragny, France) and a mixture of random hexamers and oligo(dT) primers (Promega, Charbonnieres, France). Real-time PCR assays were performed in duplicates for each sample with a Rotor-Gene 6000 (Corbett Research, Mortlake, NSW, Australia), as described elsewhere (Mazaud Guittot *et al.*, 2008). The list of the primers (Invitrogen) used in the assays is available in Table 1. Briefly, PCR was performed with 0.4 μm of each primer and 10 μL of Absolute QPCR SYBR Green ROX mix (Thermo Fisher Scientific, Courtaboeuf, France), in a total volume of 20 μL. After the initial denaturation step of 15 min at 95 °C, the reaction conditions were 40 cycles of 95 °C for 15 sec, 55 or 58 °C (depending on the primers, Table 1) for 10 sec and 72 °C for 20 sec. The fluorescence intensity of SYBR Green was read on the Rotor-Gene at the end of each extension step. Melting curve analyses were performed immediately following the final PCR cycle to verify the specificity of the PCR product by checking its Tm. Rpl19 (ribosomal protein L19), and Gusb (glucuronidase beta) genes were chosen as references for normalizing target gene**s** in the testis (Mazaud Guittot *et al.*, 2008) and liver (unpublished data from the laboratory) respectively. Relative quantification was carried out using the standard curve method for both target and housekeeping gene (endogenous control) in each sample. A series of dilutions of calibrator sample (external standard) was included in each experiment to generate an external standard curve. Then the concentration of the target gene in each sample was divided by the concentration of the housekeeping gene in each sample, normalizing the samples. Relative quantification was carried out using the lightcycler Relative quantification Software (version 1.0) (Roche Diagnostics GmbH, Mannheim, Germany). The calculation of data was based on the crossing point (Cp) values obtained by the lightcycler Software. To correct for sample heterogeneity and variability of detection, results were calculated as the target/reference ratio of the sample divided by the target/reference ratio of the calibrator.

**Table 1:** List and sequence of primers used for PCR analysis

Gene symbol	Accession no.	Forward 5′–3′	Reverse 5′–3′	Size (bp)	Topt
Ccl5/Rantes (a)	NM_031116	CTTGCAGTCGTCTTTGTCAC	GACTAGAGCAAGCAATGACAG	158	58
Ccl5/Rantes (b)	NM_031116	ACCTTGCAGTCGTCTTTGTC	ATCTATGCCCTCCCAGGAATG	224	55
Cyp1a1	NM_012540	CAAGAGCTGCTCAGCATAGTC	GCTCAATGAGGCTGTCTGTG	229	58
Glipr1	NM_001011987	TCTCTGCACTAACCCACAACG	GGAGAACTGACTTAGCGATG	124	58
Gusb	NM_017015	CTTCATGACGAACCAGTCAC	GCAATCCTCCAGTATCTCTC	117	58
Insl3	NM_053680	CTGTCTCACTGGCTGCACC	GGGTGTTTCATTGGCACAG	119	58
Pf4	NM_001007729	TTCTTCTGGGTCTGCTGTTG	ATTCTTCAGCGTGGCTATG	197	55
Rpl19	NM_031103	CTGAAGGTCAAAGGGAATGTG	GGACAGAGTCTTGATGATCTC	195	58
Sycp1	NM_012810	TTGGGAGAGGTTGAGAAAGC	CCTTTGCTGAAGACTGTTCC	205	58

The size of the expected PCR fragment in base pairs (bp) and the optimal temperature (Topt) for annealing are reported. Two reference genes were used, Gusb (Glucoronidase Beta) and Rpl19 (Ribosomal protein L19). Two couples were used for Ccl5/Rantes, Ccl5/Rantes (a) for RT-PCR and Ccl5/Rantes (b) for RT-Q-PCR.

### Isolation of testicular cells and RT-PCR

Highly enriched fractions of testicular cells were obtained as described elsewhere (Le Magueresse-Battistoni *et al.*, 1994, 1998; Longin *et al.*, 2001). Briefly, Sertoli and peritubular cells were isolated from 20-day-old rats and cultured at 32 °C in a humidified atmosphere of 5% CO_2_ in Dulbecco’s Modified Eagle’s Medium (DMEM)-Ham’s F12 (Life technologies, Grand Island, NY, USA) supplemented (peritubular cells) with or without 10% foetal calf serum. Sertoli cells were hypotonically treated to eliminate the contaminating germ cells. Enrichment of Sertoli and peritubular cells cultured for 6 days was higher than 95%. Leydig cells were isolated from adult rats as previously described elsewhere (Carreau *et al.*, 1988; Mazaud Guittot *et al.*, 2008). Briefly, following a collagenase digestion, interstitial cells were purified on a discontinuous Percoll density gradient. The interface between 40 and 60% was collected and washed to eliminate Percoll. The purity of the fraction ranged from 90 to 95%. It was assessed by the presence of 3beta-HSD activity (Bilinska *et al.*, 1997). Spermatogenic cells were isolated from adult rat testes by trypsinization. The resulting crude germ cell population (containing germ cells from all developmental steps) was submitted to centrifugal elutriation. Two fractions were harvested; the pachytene spermatocyte fraction enriched at 80–85% (contaminated primarily by early spermatids) and the early spermatid fraction (steps 1–8) enriched at 75–80% with primary contamination by both spermatocytes and elongated spermatids.

After collection, the different cell populations were processed for RNA extraction using Trizol reagent (Invitrogen, Eragny, France). RT-PCR was conducted as described (Longin *et al.*, 2001) using the primers Ccl5/Rantes (a) and Rpl19 (Table 1) to ensure equal loading in a 2% agarose gel. A DNA ladder (Promega) was loaded and gels were stained with ethidium bromide. Negative controls contained water instead of cDNA. The PCR product for Ccl5/Rantes (a) was sequenced by GENOME Express (Grenoble, France).

### Data analysis

Statistical analyses were carried out with the sigmastat 3.1 software package (Systat Software, Inc., Point Richmond, CA, USA). All values are expressed as mean ± SEM, unless specified differently. Statistical analysis was performed by anova followed by Dunnett’s test for multiple comparisons. Early bw were analysed with a two-way (dose group × day) anova model. Significance was accepted at a confidence level of *p*<0.05.

## Results

### Effect of TCDD on F1 males

Preliminary experiments were performed to determine the dose range of 2,3,7,8-TCDD. We observed that doses of 270 ng/kg bw induced maternal and foetal toxicity, and death of dams was observed at the dose of 1000 ng/kg bw (Appendix S1). Therefore, doses of 10, 100 and 200 ng/kg bw were administered to pregnant females at embryonic day 15. No treatment-related differences were noted with regard to the litter size, the sex ratio or the bw of foetuses in females that were dosed with 10, 100 or 200 ng/kg bw if data were expressed per litter. No significant interaction was evidenced between dose and age (Table 2).

**Table 2:** F1 pups and weight of the offspring

2,3,7,8 TCDD (ng/kg)		0	10	100	200
Mean number of pups/dam		12.5 ± 2.7 (9)	11.7 ± 1.5 (3)	13.7 ± 2.5 (3)	10.91 ± 2.9 (9)
Mean % of males/litter		47.6 ± 19 (9)	62.4 ± 19.2 (3)	59.2 ± 10.1 (3)	48.4 ± 13 (9)
Male pup weight (g) from 5 to 14 postnatal days	5	14.3 ± 2.4 (5)	13.7 ± 1.3 (3)	13.3 ± 1.3 (3)	12.9 ± 1.6 (5)
		13.7 ± 1.7 [32]	13.7 ± 1.3 [17]	13.3 ± 1.3 [21]	12.6 ± 1.7 (26)*
	7	20.4 ± 3.2 (5)	18.8 ± 1.8 (3)	18.0 ± 1.4 (3)	17.7 ± 2.9 (5)
		19.5 ± 2.2 [28]	18.7 ± 1.8 [19]	17.9 ± 1.4 [22]	17.2 ± 3.0 [22]*
	10	28.1 ± 4.0 (5)	26.5 ± 1.6 (3)	26.3 ± 0.8 (3)	25.4 ± 4.1 (5)
		26.9 ± 3.0 [28]	26.4 ± 2.0 [18]	26.3 ± 1.1 [22]	24.7 ± 4.3 [22]*
	12	34.9 ± 5.5 (5)	33.7 ± 1.3 (3)	32.7 ± 1.0 (3)	31.9 ± 4.5 (5)
		33.4 ± 4.1 [28]	33.7 ± 1.7 [18]	32.6 ± 1.4 [22]	31.0 ± 4.6 [22]*
	14	42.0 ± 5.6 (5)	40.6 ± 2.4 (3)	39.9 ± 1.1 (3)	38.5 ± 5.4 (5)
		40.3 ± 4.5 [28]	40.2 ± 2.6 [18]	39.8 ± 1.5 [22]	37.5 ± 5.6 [22]*

The number of dams studied is indicated in parentheses. The total number of pups weighed is indicated in brackets. Male pups were regularly weighed during the first 2 weeks. Values are mean ± SEM of (*n*) litter. For pup weight, values are mean ± SD of (*n*) litter or [*n*] pups. Statistical analysis was performed using anova, followed by multiple comparisons Dunett’s test. No significance was observed if data were expressed per litter.

* *p* < 0.05 compared with that of time-matched controls if considering data expressed per male pup.

However, if male pups at a given age of 5, 7, 10, 12 or 14 days were considered instead of litter as the experimental unit, then male pups were found to be lighter (an average of 8%; *p*<0.05) in the TCDD-200 group during the lactating period compared with the control group. No effect on weight gain was detected in rats exposed to TCDD from weaning onwards (not shown). The intra-testicular levels of testosterone and 4-androstenedione were in the normal range in 28-, 40-, 67- and 145-day-old rats of the TCDD-200 group (not shown). The other groups were not assayed. Testicular and epididymal weights were within the normal range in rats of the differently dosed groups compared with that in the control rats, and the gross histology of the testes was also normal throughout development (not shown).

### Effect of TCDD on hepatic Cyp1a1 expression levels

**Figure 1:**
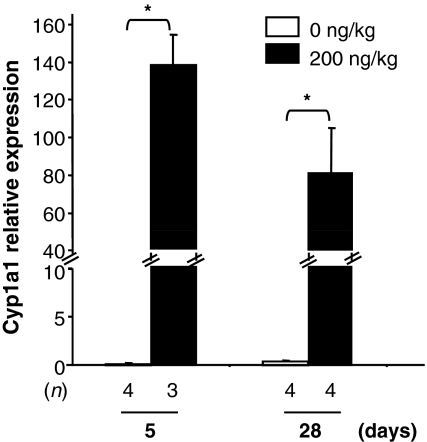
Relative expression of Cyp1a1 assessed by Q-PCR analysis in the livers of TCDD-200 male rats of the F1 generation at 5 and 28 days of age. The Gusb levels, normalized values, are the mean ± SEM of *n* = 3–4 different animals from three to four different litters. *p<0.05 compared with that of age-matched controls.

Expression of Cyp1a1 was measured by quantitative PCR in the liver of male rats aged 5 and 28 days to confirm TCDD exposure. Indeed, the induction of Cyp1a1 is regarded as one of the most sensitive end-points of AhR activation (Vanden Heuvel *et al.*, 1994). As shown in Fig. 1, expression of Cyp1a1 was dramatically enhanced in the liver of rats of the TCDD-200 group at 5 and 28 days of age (i.e. 1.5 and 5 weeks after the females had been given TCDD), probably as a result of a continued exposure of pups to dioxin through breast milk of the exposed mothers. This is consistent with the finding that the majority of TCDD in offspring of dosed dams has been shown to arise from lactational transfer of TCDD (Li *et al.*, 1995).

### Sperm counts and DSP in F1 males

Sperm counts were monitored in the caput, corpus and cauda of the epididymis of 67- and 145-day-old rats and DSP was measured at both ages (Table 3 and not shown). anova and multiple comparison Dunnett’s test were run to assess significance. No significant effect was evidenced at 145 days of age for any group or at 67 days of age for the TCDD-10 and TCDD-100 groups, regardless of whether the data were reported per litter or per rat (range 6–12). In 67-day-old rats, the *p*-value was 0.06 for sperm counts in the caput epididymis, 0.056 for sperm counts in the cauda epididymis and 0.052 for DSP when the TCDD-200 group was compared with the control group and data were expressed per litter. The *p*-value was 0.013 for sperm counts in the caput epididymis, 0.021 for sperm counts in the cauda epididymis and 0.06 for DSP, with data expressed per rat (Table 3).

**Table 3:** Epididymal sperm reserves and daily sperm production (DSP) in 67-day-old rats of the TCDD-10, TCDD-100, TCDD-200 and control groups

2,3,7,8 TCDD (ng/kg bw)		0	10	100	200
Epididymal sperm reserves (10^6^)					
Caput	Litter	48.8 ± 0.65 (9)	46.9 ± 2.3 (3)	42.4 ± 2.6 (3)	39.9 ± 4.3 (9) (*p* = 0.06)
	Rat	48.5 ± 2 (12)	47.2 ± 1.6 (7)	42.1 ± 1.5 (8)	39.5 ± 3.6 (10) (*p* = 0.013)
Corpus	Litter	7.6 ± 0.52 (9)	8.8 ± 1.7 (3)	6.8 ± 1.1 (3)	8.6 ± 1.9 (9)
	Rat	7.4 ± 0.72 (12)	8.4 ± 1.3 (7)	6.9 ± 0.7 (8)	8.3 ± 1.6 (10)
Cauda	Litter	59.9 ± 3.2 (9)	67.7 ± 1.3 (3)	59.4 ± 1.1 (3)	46.5 ± 3.4 (9) (*p* = 0.056)
	Rat	62.4 ± 3.3 (12)	70.5 ± 7.1 (7)	59.9 ± 6.4 (8)	45.9 ± 3.1 (10) (*p* = 0.021)
Daily sperm production (10^6^)					
	Litter	25.9 ± 0.9 (9)	25.2 ± 3 (3)	25.7 ± 1.8 (3)	22.5 ± 1 (9) (*p* = 0.052)
	Rat	26.3 ± 1.04 (12)	25.3 ± 1.9 (7)	25.5 ± 6.4 (8)	22.4 ± 0.9 (10) (*p* = 0.06)

Values are mean ± SEM and are expressed per litter (3–9) and per rat (7–12). Statistical analysis was performed using anova, followed by multiple comparisons Dunett’s test, and the *p*-value is reported for the TCDD-200 group compared with the control group.

### Identification of TCDD sensitive genes

**Figure 2:**
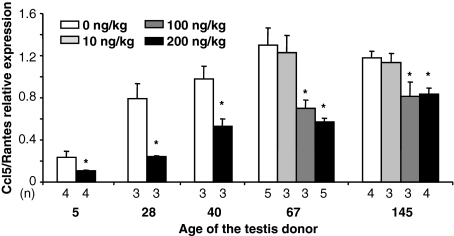
Relative expression of Ccl5/Rantes assessed by Q-PCR analysis in the testes of TCDD-200 and control rats of 5–145 days of age. Testes of TCDD-10 and TCDD-100 rats were studied at 67 and 145 days of age. The Rpl19 levels-normalized values are the mean ± SEM of *n*= 3–5 animals from three to five different litters. *p<0.05 as compared with that of controls.

**Figure 3:**
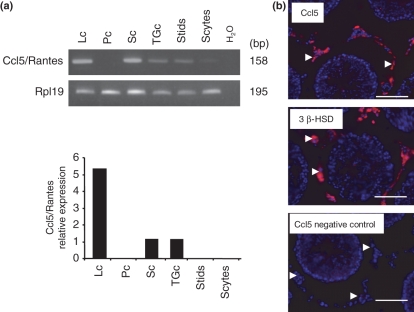
Identification of the testicular source of Ccl5/Rantes. (a) Total RNA was extracted from 6-day-old cultured peritubular cells (Pc) or Sertoli cells (Sc) recovered from 20-day-old rat testes; from a fraction enriched in Leydig cells (Lc), a crude fraction of germ cells (TGc), elutriated fractions enriched in spermatids (Stids) or in pachytene spermatocytes (Scytes) (all from adult rat testes). RT-PCR analysis was conducted on four independent series of samples, and one representative series is shown. Rpl19 was used to estimate roughly the differences of expression between samples. (b) Immunohistochemical localization of Ccl5/Rantes (red labelling) on testis paraffin sections. Staining for 3β-HSD (red labelling) was used to identify Leydig cells in the interstitium. Leydig cells were strongly labelled for Ccl5/Rantes in contrast to seminiferous tubules, which were weakly labelled. Nuclei are blue-labelled (DAPI staining). Arrowheads point to Leydig cells. Control (−) for Ccl5/Rantes indicates that the primary antibody against Ccl5/Rantes was omitted. Bar: 100 μm.

Microarray analysis was developed using 28-day-old rat testes RNA. A restricted number of genes were selected. Specifically, three genes were up- and five genes were down-regulated by ±1.40-fold in testes of males from TCDD-200 dosed dams compared with that in control testes (SAM procedure with FDR <5%). Four genes have been reported to exhibit xenobiotic response elements (XRE; http://www.drgap.nies.go.jp/pub/page/element) in the 5′ flanking regions of the *Mus musculus* orthologous genes (Table 4). These genes were: Insl3 produced by Leydig cells, the tumour suppressor gene Glipr1 and 2 chemokines Cxcl4 and Ccl5/Rantes. They were selected and further studied by Q-PCR (Table 4). Insl3 and Cxcl4 gene expression levels, which were up-regulated at 28 days of age, were found not to be statistically different in TCDD-200 vs. control testes at the other ages studied (5, 67, 145 for lnsl3 and 67 and 145 for Cxcl4; not shown). Glipr1 gene expression levels were significantly (*p*<0.05) enhanced at 28 (Table 4) and 67 days, but not at 5 days of age (not shown). Ccl5/Rantes gene expression levels were down-regulated throughout development in TCDD-200 vs. control testes (Fig. 2). In control testes, gene expression levels of Ccl5/Rantes increased as a function of age and a plateau value was reached at 67 days of age. In TCDD-200 testes, Ccl5/Rantes gene expression levels also increased as a function of age. However, levels were significantly (*p*<0.05) decreased in TCDD-200 testes compared with that in the age-matched controls. Specifically, levels in TCDD-200 testes represented 45, 31, 54, 44 and 71% of the control levels at 5, 28, 40, 67 and 145 days of age respectively (Fig. 2). We also surveyed Ccl5/Rantes levels in TCDD-10 and -100 testes from rats of 67 and 145 days of age. No effect was observed in TCDD-10 testes, and TCDD-100 testes exhibited levels of Ccl5/Rantes of the same range as that in TCDD-200 testes (Fig. 3). Data on RNA levels could not be extended to the protein level because the signal for Ccl5/Rantes was barely detectable using Western blot analysis (not shown).

**Table 4:** List of genes up- and down-regulated in 28-day-old rat testes from the TCDD-200 group

Gene symbol and known function or localization		Accession number	Micro array fold change	Q-PCR fold change (*n* = 3)	Number of 5’ flanking XRE in mouse orthologous genes
Insl3	Leydig cells	NM_053680	1.49	1.41 ± 0.10	6
Glipr1	Tumour suppressor	NM_001011987	1.45	1.65 ± 0.10	1
Cxcl4	Chemokine	NM_001007729	1.39	1.66 ± 0.24	2
Q4V7D5		XM_576624	−1.48	nd	nd
RGD1561017		XM_577094	−1.53	nd	nd
RT1-CE5 variant 1, variant 2		NM_001008843, NM_001033986	−1.53	nd	nd
LOC679900		XM_574899	−1.83	nd	nd
Ccl5/Rantes	Chemokine	NM_031116	−1.86	−2.70 ± 0.05	3

nd, not defined; XRE, xenobiotic response elements.

Data for Q-PCR are mean ± SEM of three testis samples from three different litters.

### Identification of the testicular source of Ccl5/Rantes

Using RT-PCR analysis and specific primers for Ccl5/Rantes, a PCR product of the right size and sequence (not shown) was detected in Leydig cells, and to a much lower degree in Sertoli and germ cells (Fig. 3a). To extend these data, we found that the Ccl5/Rantes immunoreactivity was confined to interstitial cells positive for 3beta-HSD identifying the Leydig cell population (Fig. 3b). Sections incubated without the primary Ccl5/Rantes antibody remained unstained (Fig. 3b).

### Reproductive performance of F1 males and the F2 generation

F1 males from the control and TCDD-200 groups were mated with two virgin females per male during postnatal week 15. Pregnant females did not receive TCDD during gestation. All females became pregnant and no differences in the outcome of pregnancy, litter size and sex ratio were recorded between groups (Table 5). No difference in male pup weight was detected between groups, hepatic Cyp1a1 gene expression levels remained at nadir in both groups and Ccl5/Rantes mRNA testicular levels measured in 67- and 145-day-old rats were in the same range in both groups. Sperm counts and DSP were also in the normal range in male rats of the F2 generation, in both groups (Table 5). Expression per rat instead of per litter did not change the significance of the data in Table 5 (not shown).

**Table 5:** Generation of F2 males’ raw data of end-points surveyed

End-points surveyed		Control	TCDD 200 ng/kg bw
No. males		8	7
No. dams		16	14
Mean number of pups/dam		13.19 ± 3 (16)	12.64 ± 3 (16)
Mean % of males/litter		51.6 ± 12.2 (16)	47.2 ± 13 (16)
Male pup weight (g)	5 days	12.7 ± 1.1 (8)	13.9 ± 2.2 (7)
		12.6 ± 1.4 [51]	13.2 ± 1.9 [30]
	8 days	19.9 ± 1.7 (8)	20.2 ± 3.9 (7)
		19.7 ± 2.4 [51]	19.2 ± 3.7 [30]
Q-PCR data (relative expression)			
Hepatic cyp1a1 levels	20 days	2.7 ± 3.2 (3)	1.6 ± 1.9 (3)
Testicular Ccl5/Rantes levels	67 days	0.84 ± 0.09 (4)	1.06 ± 045 (3)
	145 days	1.05 ± 0.13 (4)	1.31 ± 0.23 (3)
Daily sperm production (10^6^)	67 days	22.7 ± 1.02 (4)	25.3 ± 4.1 (3)
	145 days	31.3 ± 3.4 (4)	31.4 ± 1.3 (3)
Epididymal sperm reserves (10^6^)	67 days	73.5 ± 9.7 (4)	95.5 ± 9.8 (3)
	145 days	244.9 ± 29.2 (4)	261.5 ± 12.9 (3)

The number of dams studied is indicated in parentheses. The total number of pups weighed is indicated in brackets. Values for pup weight are the mean ± SD of (*n*) litters or [*n*] pups. Data were not significant. Q-PCR data, DSP and sperm reserves are expressed as mean ± SEM of (*n*) samples, each sample originated from a different litter.

## Discussion

In this study, we demonstrated that exposure of female rats to TCDD 200 ng/kg bw at embryonic day 15 induced decreased sperm reserves in the male offspring at 67 days, but not at 145 days of age. Gene expression profile revealed that Ccl5/Rantes, a chemokine almost exclusively found in Leydig cells, was negatively regulated in testes of males from exposed dams. No such phenotype was evidenced in males of the subsequent generation.

Many studies using rats as experimental models reported the use of maternal doses of up to 1000 ng/kg (Mably *et al.*, 1992; Gray *et al.*, 1995; Sommer *et al.*, 1996; Cooke *et al.*, 1998; Haavisto *et al.*, 2006; Adamsson *et al.*, 2008). However, in this study, we observed maternal and foetal toxicity at the dose of 270 ng/kg, and death of dams with the dose of 1000 ng/kg. Therefore, to avoid toxic effects that would render the interpretation of our studies difficult, doses of 10, 100 and 200 ng/kg were used in this study. In line with a previous study (Bell *et al.*, 2007a), we observed that neonates from TCDD-200 exposed dams (if considered individually) were about 8% lighter than pups born from control dams during the lactating period. It has been demonstrated that the majority of occurrences of TCDD in offspring of dosed dams arise from lactational transfer of TCDD (Li *et al.*, 1995). Further studies will have to be addressed to determine if breast milk was less nourishing/abundant. It is also possible that TCDD interfered with food intake (Tuomisto *et al.*, 1999). In addition, we found no alteration of the sex ratio, which is in agreement with recent studies (Rowlands *et al.*, 2006; Bell *et al.*, 2007a).

Maternal exposure to TCDD leads to controversial data regarding sperm reserves in the male offspring, as stated in the Introduction. One explanation for this debated question may reside in the use of different strains, as it is known that rats may be differentially resistant to TCDD exposure (Simanainen *et al.*, 2004). In this study, we showed a significant decrease in caput and cauda sperm reserves of the TCDD-200 group at 67 days of age if data were expressed per rat. The origin of the reduced production of sperm cells in the young adult rats is currently unknown. Indeed, the decline was transitory, i.e. these parameters were in the normal range in 145-day-old rats from TCDD-200 exposed dams. Gross histology of the testis and number of apoptotic germ cells, as well as intra-testicular testosterone levels and epididymal and testes weights, were all in the normal range compared with that of the controls. In addition, dams were dosed at embryonic day 15, at the onset of testicular testosterone production. Considering that TCDD half-life is close to 3 weeks in rat (Bell *et al.*, 2007b), it implies that foetuses were maximally exposed in the first few days following dosing, i.e. during the early programming critical window for masculinization of the reproductive tracts in rat (Welsh *et al.*, 2008). Collectively, these observations would suggest that the androgenic signalling pathway was not targeted by a prenatal TCDD exposure in postnatal rats, which is consistent with previous studies (Gray *et al.*, 1995; Haavisto *et al.*, 2006).

Gene expression profiles were used to select differentially expressed genes which could be regarded as markers of a dioxin exposure. We used 28-day-old rat testes because at that age, spermatids massively populate the tubules, and first elongated spermatids are differentiated at 28 days of age. Germ cells are not over-represented, thus allowing the identification of a potential defect in somatic cells. In addition, hepatic Cyp1a1 levels were highly induced at 28 days of age, indicating that at this age TCDD was still exerting a direct genomic action. Using RNAs extracted from testes of the TCDD-200 group, we selected a restricted number of genes with fold changes of ±1.40. Three genes were up-regulated and five genes were down-regulated. Four genes were further selected based on their identity and the presence of XRE in their flanking regions, indicating that they might represent direct target genes. The up-regulated Glipr1 gene has been found to exert tumour suppressor functions and is a p53 target gene (Li *et al.*, 2008). In addition, it has previously been identified as a dioxin-sensitive gene in human subjects (McHale *et al.*, 2007). Further studies will be required to identify if its up-regulation in 28- and 67-day-old rat testes from the TCDD-200 group could contribute to the decline in DSP and sperm reserves observed at 67 days of age.

Insl3 is involved in testicular descent during embryo development (Nef & Parada, 1999), and has been suggested to act as a pro-survival factor in germ cells of adult testes (Kawamura *et al.*, 2004). Therefore, it might be speculated that the up-regulation of Insl3 would counterbalance deleterious effects induced by TCDD. However, expression levels were in the normal range in adult rats of exposed dams. In addition, expression levels at 5 days of age were very low, resulting in high variations in the Q-PCR dosage, and lack of significance.

The two other genes validated by quantitative PCR were the chemokines Cxcl4 and Ccl5/Rantes. Chemokines have previously been identified as dioxin-sensitive genes in a microarray analysis of adipocytes treated with TCDD in vitro (Hanlon *et al.*, 2005). Cxcl4, which was up-regulated at 28 days of age but not at the older ages investigated, was not further studied. Ccl5/Rantes was expressed as a function of age and down-regulated in the testes of males from TCDD-200 dosed dams throughout development. It has previously been reported in the testis (Le Goffic *et al.*, 2002), and we identified Leydig cells as the major site of Ccl5/Rantes expression. Leydig cells are also a target of the chemokine because specific receptors were detected through RT-PCR (DR, EC and BLMB, unpublished observations).

Rantes (for ‘regulated upon activation normal T cell expressed and secreted’), now given the immunological designation Ccl5, was originally identified as a typical chemokine as it was able to recruit leucocytes to the sites of inflammation. From its discovery, it was found that in addition to T cells, Ccl5/Rantes is produced by many other types of cells, including fibroblasts, endothelial and epithelial cells. Moreover, its activity is not merely restricted to chemotaxis. Beneficial activities have been described, including antimicrobial and antiviral as well as detrimental effects. For example, Ccl5/Rantes enhances inflammatory processes and has been associated with the induction or promotion of cancer (Appay & Rowland-Jones, 2001; Levy, 2009). Interestingly, Ccl5/Rantes is present in both the male and female genital tract fluids and spermatozoa exhibit specific receptors for the chemokine. However, consistent with high levels found in diseases related to infertility including genital tract infections, Ccl5/Rantes has been shown to impact sperm fertilizing ability negatively (Barbonetti *et al.*, 2008). Therefore, the decrease in Ccl5/Rantes gene expression levels observed in this study in testes of rats born from exposed dams could be part of a protecting mechanism against TCDD impact. It remains to determined if (and to which extent) decreased Ccl5/Rantes gene expression levels contributed to the transitory sperm count decline. For example, sperm counts were normal in 145-day-old rats, whereas Ccl5/Rantes gene expression levels were significantly lower than that of controls at that age. In addition, we may have bypassed important genes impacted as well by a TCDD exposure, and contributing with or independently of Ccl5/Rantes in decreasing sperm counts. A transcriptomic study using testes of 67-day-old rats may be useful to answer this point.

Inhibition of Ccl5/Rantes gene expression levels exerted by dioxin was observed 20 weeks after dams were given either 100 or 200 ng/kg bw TCDD, and 16 weeks after weaning if considering that the majority of TCDD in offspring of dosed dams has been shown to arise from lactational transfer of TCDD (Li *et al.*, 1995). As TCDD half-life is close to 3 weeks in rats (Bell *et al.*, 2007b), it is possible that increased transcriptional activity coupled to mRNA stability could account for the persisting decreased effects observed at 67 and 145 days of age. Further studies using primary cultures of Leydig cells are warranted to fuel this hypothesis.

Several studies have provided evidence that endocrine disrupters might cause epigenetic alterations which could be transmitted to subsequent generations through the male germ line (Anway *et al.*, 2005) or the maternal lineage (Newbold *et al.*, 2006). We thus examined the reproductive performance of the F1 males from TCDD-200 dosed dams, and F3 males were also generated using F2 males (not shown). Interestingly, we observed that sperm reserves, DSP and testicular Ccl5/Rantes gene expression levels were in the normal range in the F2 and F3 (not shown) generations. However, because exposure was acute and performed at embryonic day 15, at the onset of a common programming window in which androgen action is essential for normal reproductive tract masculinization (Welsh *et al.*, 2008), but a few days after reprogramming of the germ line has occurred (Sasaki & Matsui, 2008; Trasler, 2009), chronic dosing studies covering the whole developmental period should be carried out before one could conclude the lack of transgenerational effect of dioxin.

In conclusion, our data demonstrated that maternal exposure to TCDD at doses of 100 and 200 ng/kg bw impacted the reproductive function of the F1 males. We propose that the chemokine Ccl5/Rantes might be regarded as a marker of TCDD exposure. Future studies will help to determine to which extent Ccl5/Rantes in Leydig cells may regulate spermatogenesis. In addition, given that TCDD has been shown to exhibit various endocrine disrupting effects, interacting with both the oestrogenic and androgenic pathways (Ohtake *et al.*, 2003), it would be of interest to determine whether Ccl5/Rantes is modulated as well by chemicals with oestrogenic and/or antiandrogenic activities.

## Supporting information

**Appendix S1.** Description of the two preliminary experiments settled up to determine the dose-range of TCDD.
