# Supplementary material for: The effects of an in utero exposure to 2,3,7,8-tetrachloro-dibenzo-p-dioxin on male reproductive function: identification of Ccl5 as a potential marker
Source: Int J Androl. 2010 Apr;33(2):413–24. doi: 10.1111/j.1365-2605.2009.01020.x (PMC2871170; doi:10.1111/j.1365-2605.2009.01020.x)
Supplement: Supplementary file 1 [file ija0033-0413-SD1.doc]

Supplemental Data: Description of the 2 preliminary experiments settled up to determine the dose-range of TCDD

Experiment 1: Four groups of five dams each were orally treated with 0, 27, 140 or 270 ng/kg bw. Timing of dosing was Embryonic Day 11. Animals were killed at different developmental ages. Intra-testicular content of testosterone and 4-androstenedione were performed in samples collected at different periods of development. We also performed histological analysis and fertility tests. Sperm reserves were counted in animals aged of 159 days. No treatment-related differences were noted with regard to the outcome of gestation, the litter size, the sex-ratio or the body weights of fetuses from females that were dosed with the two lower doses of TCDD. However, one dam had aborted and a second one gave birth to 10 dead fetuses in the group administered the highest dose. We did not collect samples from the dead fetuses, and the origin of their death has not been investigated. The gross histology of the in utero exposed rat testes of the different TCDD dosed groups including the highest dosed-group was comparable to the control group. Intra-testicular hormone levels in the treated animals were also in the normal range. Animals could reproduce and sperm reserves assessed at 159 days of age were in the normal range.

Experiment 2: A unique dose of TCDD of 1000 ng/kg bw was administered at Embryonic Day 15 to mimic the design of previous experiments leading to a spectrum of effects in the reproductive system of the male offspring (Ref. in Introduction). Two groups of 5 dams each were handled. The five control dams had normal gestation, normal litter size and sex-ratio. In the TCDD-dosed dams group, one dam died before giving birth, 2 dams had eaten their fetuses soon after delivery, one dam gave birth to one male, and one dam gave birth to one male and two females. The four pups died a few days after birth. The three surviving dams were killed showing apathy and stuck and bristly hair.

Aliquots containing various doses of dioxins were assayed by Dioxlab (Dioxlab, 94417 Saint-Maurice) to ascertain doses given to the animals. Therefore, maternal and foetal toxicity were observed in our hands at the dose of 270 ng/kg bw, and death of dams occurred at the dose of 1000 ng/kg bw.
